# Single shot laser writing with sub-nanosecond and nanosecond bursts of femtosecond pulses

**DOI:** 10.1038/s41598-017-16850-z

**Published:** 2017-11-29

**Authors:** Andrey Okhrimchuk, Sergey Fedotov, Ivan Glebov, Vladimir Sigaev, Peter Kazansky

**Affiliations:** 1 D. Mendeleyev University of Chemical Technology of Russia, Miusskaya square 9, Moscow, 125047 Russia; 2 Optoelectronics Research Centre, University of Southampton, Southampton, SO17 1BJ UK

## Abstract

A method is proposed for efficient laser modification of fused silica and sapphire by means of a burst of femtosecond pulses having time separation in the range 10–3000 ps. Modification enhancement with the pulse separation increase in the burst was observed on the tens picoseconds scale. It is proposed that accumulated transient tensile strain in the excitation region plays a crucial role in modification by a sub-nanosecond burst.

## Introduction

Femtosecond laser direct writing and micromachining attract considerable interest for a variety of applications ranging from integrated optics and microfluidics to printable flat optics and multi-dimensional optical data storage. A regular train of femtosecond pulses generated by a femtosecond oscillator or regenerative amplifier is commonly used in laser material processing. Micromachining with temporally shaped pulses or femtosecond pulse trains exploits material dependent electronic relaxation processes. It has been applied, for example, to creating sub-wavelength features via control of ionization processes on a sub-picosecond time scale^1,2^. Control of heat deposition with sub-microsecond bursts of femtosecond pulses permits the of writing of waveguides with a circular cross-section^3,4^.

In addition to heating, the creation of a pressure wave and rarefaction in the modified zone are other important factors determining laser induced structural changes^5^. The strain develops nearly 100 times quicker than the accumulation of heat; that is, on a sub-nanosecond scale. Probably this is the reason why stress and strain have not yet been used in the control of laser processing of materials.

In the heat accumulation regime the simplest burst generation technique is based on pulse picking from a continuous train of pulses^3^. The interval between pulses in a burst is defined by the femtosecond laser repetition rate and cannot be less than some tens of nanoseconds. A more sophisticated setup allowing control of the energy of each pulse in the burst makes use of a passive resonator with Pockels cell^4^. With such a setup the pulse separation interval obviously cannot be reduced to less than around 10 ns, because reduction of the cavity length is limited by the space occupied by the Pockels cell inside the cavity. To our knowledge there are no laser writing setups allowing the generation of bursts with pulse separation intervals in the range 10–10000 ps. Accordingly, the interaction of focused femtosecond pulses with materials in the regime of such bursts has not yet been investigated.

An enhancement of the strength of nanograting induced birefringence was observed upon writing with a train of double pulses, when the interval between pulses was less then 50 ps, presumably due to increased absorption of self-trapped excitons (STE)^6^. An enhanced change in refractive index was obtained in a waveguide written by a train of double pulses separated by intervals in the range 400–800 ps^7^. This last observation has been attributed to additional absorption by colour centres, assumed to be a product of STE relaxation. This in turn could suggest that a burst of pulses, separated by a few tens or hundreds of picoseconds, would produce material modification greater than modification produced by a single pulse with the same energy.

In this paper we will propose and demonstrate a method for efficient micromachining of silica glass and sapphire by means of sub-nanosecond bursts of femtosecond pulses with decaying amplitudes in each burst. We show that a burst with pulse separation interval in the range 10–3000 ps activates an additional mechanism of femtosecond modification based on simultaneous actions of the laser pulse and rarefaction or tensile strain, produced by the cumulative action of previous laser pulses in the burst. Contrary to common belief, we observed an increase in refractive index change with increase of time separation between pulses in the burst on a time scale of 0–100 ps, which we connect with the setting time of tensile strain in the modifying region.

## Methods

### Experimental setup for femtosecond modification

Transform-limited pulses with a duration of 180 fs were generated by an ytterbium solid-state laser system at 1030 nm. A high precision translation stage (Aerotech ALB1000) was set up to move the sample – either a fused silica plate or a z-cut sapphire plate – between laser shots and perpendicularly to the beam. An Olympus objective lens (NA = 0.65) was used to focus the laser beam, with correction of spherical aberrations, into the sample. We investigated the modifications and non-linear absorption of pulses arising in a single shot regime. That is, either a single pulse or burst of pulses was focused on a pristine region of a sample. For the silica experiment, the laser beam was focused so that the beam-waist was located at a depth of 150 μm. For the sapphire experiment, the laser beam was focused at a depth of 90 μm.

**Figure 1:**
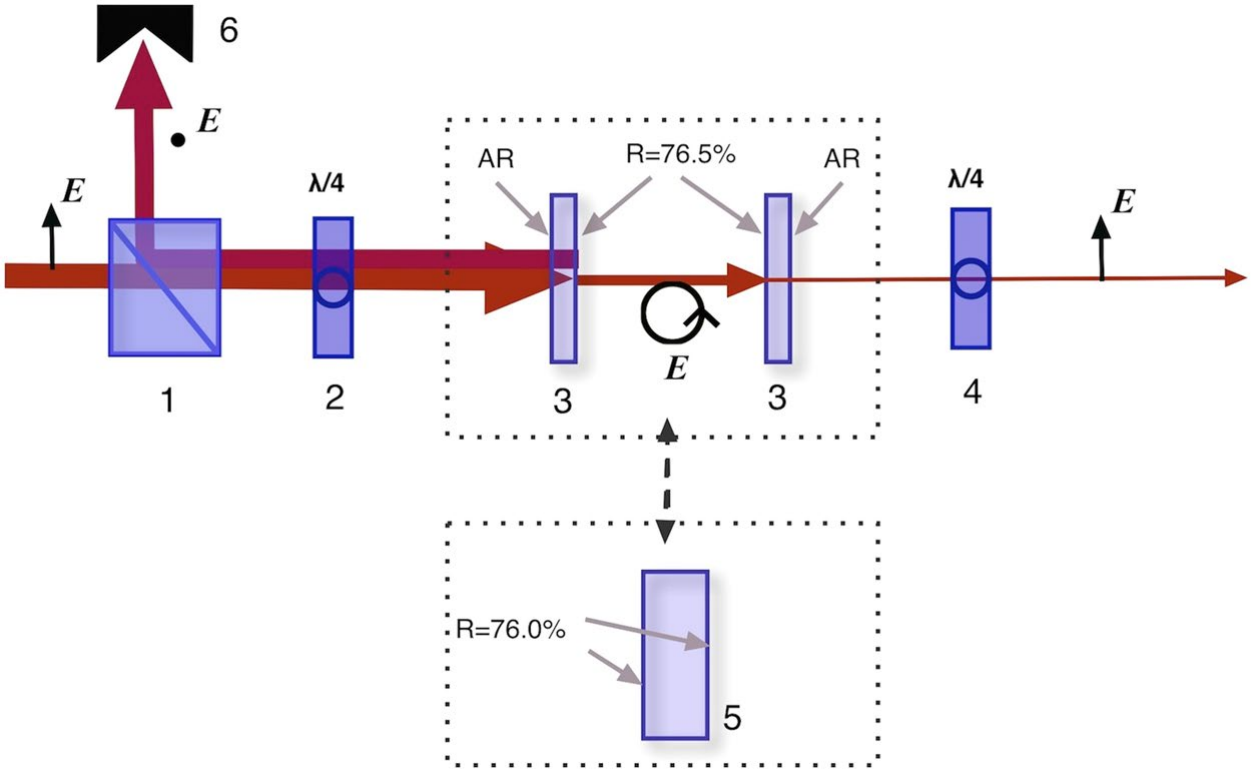
Schema of the optical setup for generation of a burst of pulses. 1- Glan-Taylor prism; 2, 4 - quarter-wave plates; 3 – semi-transparent mirrors of the adjustable Fabri-Perot cavity for burst generation with inter-pulse separation *Δt* of 70 ps–8 ns; 5 – monolithic Fabri-Perot cavity for burst generation with inter-pulse separation *Δt* in the range 10–70 ps; 6 – beam stop; ***E*** denotes direction of polarization.

In order to produce pulse bursts with various different inter-pulse separations, we used two different kinds of Fabry-Perot cavities (Fig. 1). (1) To generate bursts with longer inter-pulse separation, we used a single adjustable Fabry-Perot cavity made using two plane semi-transparent dielectric mirrors. Depending on the cavity length, this cavity can be adjusted to produce bursts with inter-pulse separation Δ***t*** in the range 70 ps–8 ns. On the interior side the mirrors had a partially reflecting chirp-free dielectric coating with reflection coefficient of 76.5%, and on the exterior side an AR coating designed for wavelength of 1030 nm. (2) To generate bursts with shorter inter-pulse separation, we used a monolithic Fabry-Perot cavity consisting of a single plane-parallel fused silica plate, with sides parallel to within 2 arc seconds, reflection coefficient of 76.0%, and thicknesses varying from 1 to 7 mm. Depending on the plate thickness, this second type of cavity produces bursts with inter-pulse separation Δ*t* in the range 10–70 ps.

Precision of the experimental data depends critically on the precision with which the Fabry-Perot cavity is set up. For a burst with Δ*t* between 10 and 70 ps, a suitably chosen monolithic cavity is positioned in the path of the laser beam in such a way that the back reflected beam overlaps with the incoming beam, and no further adjustments are made. For bursts with longer inter-pulse separations, using an adjustable cavity of the first type, the first step is to position the first mirror of the cavity in the beam path so that the back reflected beam overlaps with the incoming beam. Next, a laser beam profiler is placed behind the cavity, and the second mirror of the cavity is tuned in such a way that the envelope profile of the laser beams leaving the cavity be as close as possible to a Gaussian profile of the original laser beam. For all cavities, we were able to ensure that all the burst pulses were focused into the same region of the sample, with an error estimated to be less than 100 nm. Despite this high degree of precision, the unavoidable instability inherent in the original laser beam as well as that due to air fluctuations both produce variation in the beam direction, and this effect becomes significant for cavities longer than half a meter, corresponding to bursts with a inter-pulse interval Δ*t* of more than 3 ns. An optical isolator consisting of a quarter-wave plate and a Glan-Taylor prism polarizer was included to prevent possible optical damage of the laser system by energetic pulses reflected back from a cavity (Fig. 1, positions 1 and 2). Another quarter-wave plate was set after the cavity to change the polarization back from circular to linear (position 4). A half-wave plate not shown in Fig. 1 controlled the direction of linear polarization of the beam hitting the sample.

**Figure 2:**
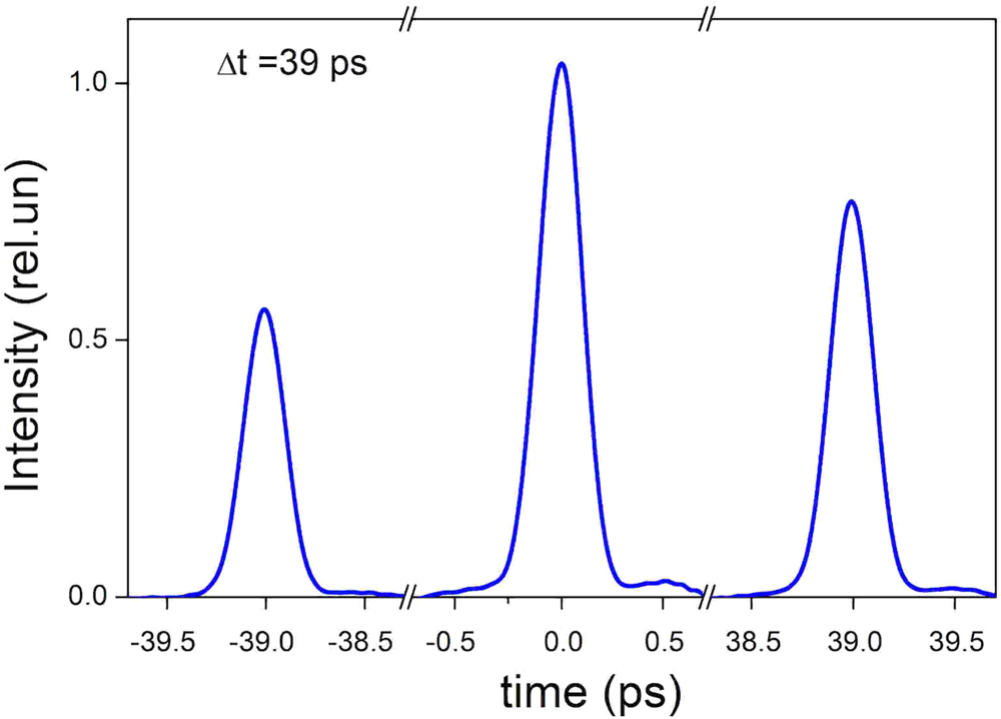
Autocorrelation function of the burst produced by the monolithic Fabri-Perot cavity of thickness 4 mm. The central peak corresponds to small delays between arms of the autocorrelator when each pulse in the burst interacts with itself, and the side peaks correspond to large delays when each pulse in the burst interacts with its neighbour.

The duration of pulses in the burst was checked with a scanning autocorrelator (Fig. 2). We performed measurements of autocorrelation functions of the burst with pulse separation interval of 39 ps in two regimes: (1) with nearly equal autocorrelator arms; (2) with bias difference between lengths of the arms giving time delay equal to the pulse interval. In the first case we obtained autocorrelation in such a way that each pulse in the burst interacted with itself, and in the second case each pulse interacted with a neighbouring pulse in the burst. In both cases the shape of the autocorrelation function coincided with the autocorrelation function for an original pulse coming from the laser system, and the calculated duration of a separate pulse is equal to 180 fs assuming Gaussian pulse shape. This result shows that the Fabry-Perot cavity affected neither duration nor shape of each pulse in the burst.

In the described experimental setup a sample was exposed to a train of pulses with identical shape but with exponentially decreasing energy from each pulse to the next. Assuming exponential decay of pulse energy in the burst, according to calculations, the energy of the first pulse, the highest within the burst, was less by a factor of 2.4 than the energy of the whole burst. With the above technique it is possible to obtain 10–1000 times longer effective burst duration compared to the duration of a tailored pulse produced by a third-order chirp without stretching each pulse in the burst^1^.

In order to investigate the dependence of energy deposition in fused silica on the pulse separation interval we measured the dependence of the non-linear transmittance of a glass plate on the incoming energy of the original pulse or the burst of pulses *E*_*in*_ for different pulse intervals. A photodiode sensitive head measured the reference energy of the burst or pulse, and another measured the energy passed through the sample. A sensitive head was placed as close as 3 mm behind the sample, and the diameter of its sensitive area was as high as 10 mm, and was large enough to collect more than 95% of the energy passing through the sample. The laser system operated at a repetition rate of 1 kHz, and the sample was moved perpendicular to the laser beam at a speed of 2 mm/s. Thus the transmittance of each burst or pulse was measured in a pristine area of the sample (modified spots were separated by 2 μm). The transmittance data were collected and stored on a computer.

### Characterization of modifications

Refractive index change and birefringence in the laser modified point regions were investigated with a bright field microscope Olympus BX61 operating in transmitting mode, equipped with a 14-bit CCD camera, motorized object table and quantitative birefringence imaging system (CRi Abrio Imaging System).

In the first step, mapping of the optical path *φ(x*,*z)* was obtained by mathematical processing of bright field images with quantitative phase microscopy software (QPM)^8^. The images were taken and a corresponding QPM treatment was carried out for two viewing directions, one along the direction of propagation of the writing laser beam (top view), and the other perpendicular to it (side view). Three bright field images were captured to calculate one mapping of the optical path. One image was taken with the translation stage in the focus position and the other two images were taken with the translation stage moved, with accuracy of 0.1 μm, to positions defocused by ±1μm. The bright field images were taken with an interference filter transmitting at 503 nm with bandwidth of 10 nm. The objective lens had NA = 0.45 for the top view and NA = 0.75 for the side view, and the condenser diaphragm was set at NA = 0.2. The spatial filter was set to 2.0 during the QPM analysis.

The 3D distribution of the refractive index in modifications made in silica glass was calculated by use of the inverse Abel transform. It was assumed that modified points have axial symmetry relative to the direction of the laser beam. By imposing such a symmetry assumption, the optical path mapping observed in the direction perpendicular to the laser beam (side view) can be presented by the Abel transform^9^:

1 $$\phi (x,z)={\phi }_{0}+2{\int }_{x}^{\infty }\frac{{\rm{\Delta }}n(r,z)rdr}{\sqrt{{r}^{2}-{x}^{2}}},$$

where *φ(x, z)* is optical phase for unmodified region of glass. The inverse Abel transform yields the radial dependence of change in the refractive index, following measurement of optical path distribution:

2 $${\rm{\Delta }}n(r,z)=-\frac{1}{\pi }{\int }_{r}^{\infty }\frac{\partial \phi (x,z)}{\partial x}\frac{dx}{\sqrt{{x}^{2}-{r}^{2}}}.$$

In practice it is convenient to approximate the optical path as a Fourier cosine series in the coordinate X representing displacement perpendicular to the symmetry axis^9,10^:

3 $$\phi (x,z)={a}_{0}(z)+\sum _{m=1}^{M}{a}_{m}(z)\cos (\frac{m\pi x}{R}),$$

where *R* is the radius of the treated region, which is of cylindrical form. The change in refractive index is negligible at the boundary of this region. *M* is the number of terms in the sum that are necessary for a good approximation and was taken to be equalled to 15. Integration of formulas (3) and (2) gives a formula for calculation of the change in the refractive index:

4 $${\rm{\Delta }}n(r,z)=\sum _{m=1}^{M}\frac{m{a}_{m}(z)}{R}{\int }_{r}^{R}\frac{\sin (\frac{\pi mx}{R})}{\sqrt{{x}^{2}-{r}^{2}}}dx.$$

## Results

### Refractive index change and optical path difference

All laser-writing experiments were done in a single shot regime with each single pulse or single burst affecting a new pristine region in the sample.

We began by comparing the effectiveness of a single pulse and a burst with pulse separation of 70 ps. To do this we proceeded incrementally in steps of 5 nJ from an energy of 10 nJ up to 200 nJ, to locate the threshold energy at which inscription takes place. At each step the exposed region of the sample was analysed using the QPM technique. It was found that the threshold energies for a single pulse and for a burst, under our experimental conditions, were close to 30 nJ and 75 nJ respectively.

**Figure 3:**
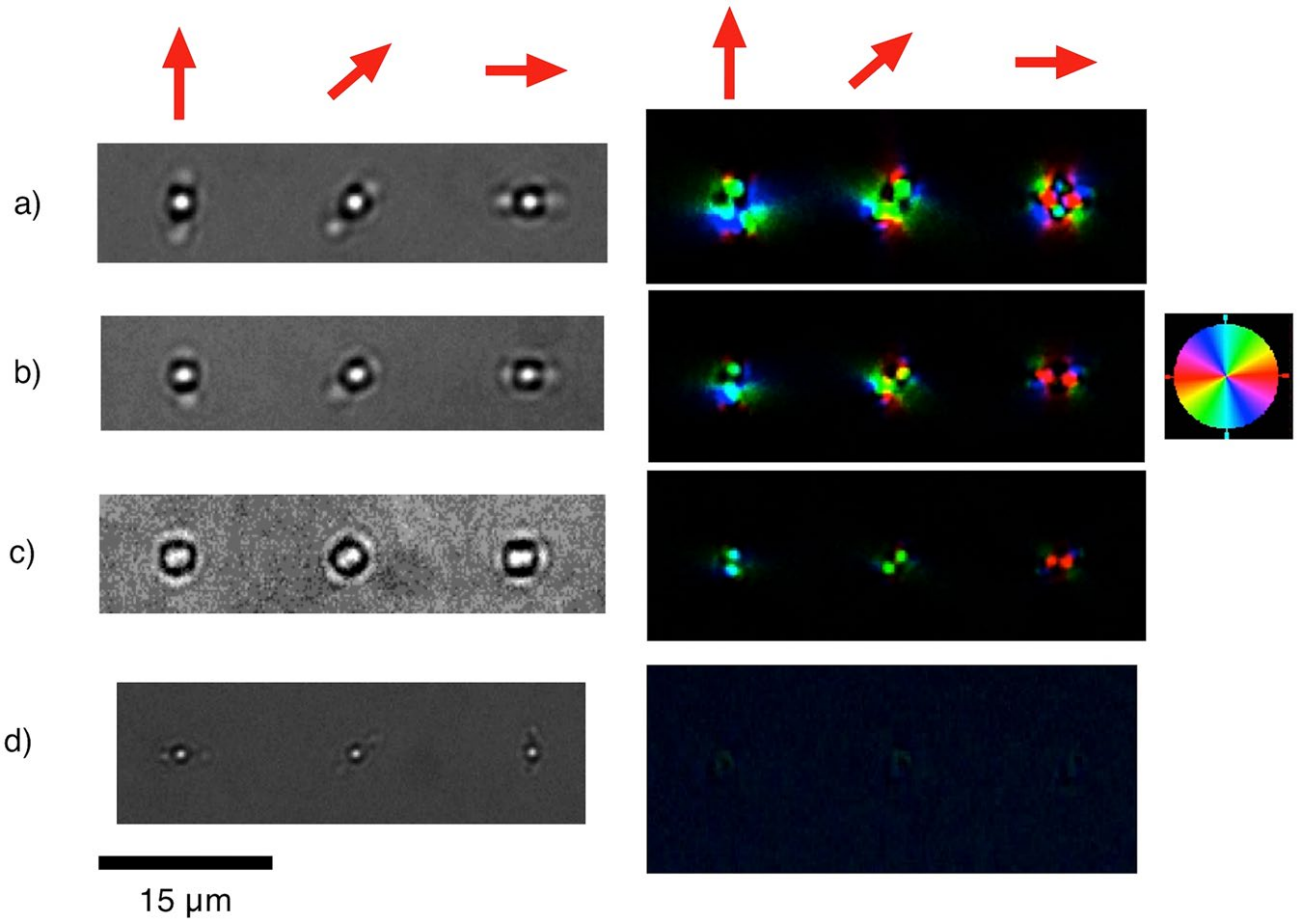
Bright field microscopic pictures (on the left) and mapping of the induced birefringence (on the right) produced in silica glass by a burst with pulse interval of 10 ps (**a–c**) and with a single pulse (**d**) for three different linear polarization directions, indicated by red arrows. The burst energies are: (**a**) 225 nJ, (**b**) 112 nJ, (**c**) 90 nJ, the single pulse energy (**d**) is 99 nJ. The colours code different directions of the slow axis of birefringence, as represented in the small square, with legend included on the right. Brightness codes retardance.

Next, we investigated writing with a single pulse and with bursts having various pulse separation intervals for energies in the range of 80–200 nJ. Microscopic bright field pictures for fused silica taken in the direction of the laser beam (top view) are presented in Fig. 3. The shape of the modifications differs slightly from a circular form, that is, it has “ears” on opposite sides of the central modification, and the orientation of the ears coincides with the polarization of the writing beam. The ears are rather weak in the spots produced by a single pulse, and they are more pronounced in the modification made by a burst, especially at higher energies. They are almost indiscernible for a burst with energy of less than 90 nJ (Fig. 3c). Overall sizes of the spots produced by a burst are larger than those produced by a single pulse.

It was found that, contrary to what happens with a single pulse, a burst produces a birefringent microstructure in fused silica. Two birefringent spots form a dumbbell-like structure with the slow axis coinciding with the line connecting the spots, while the region between the spots remains birefringence free (Fig. 3). Orientation of the dumbbell structure and of the birefringence slow axis both follow polarization of the femtosecond beam in such a way that the slow axis and the line connecting the birefringent spots are both parallel to the polarization. The energy threshold for producing the birefringence structure coincides with the modification threshold, and the structure is at its most well-defined just above the modification threshold with retardence at the centres of the spots around 10 nm, as can be seen in Fig. 3c. At higher burst energies, stress around the structure presumably destroys spatial uniformity of the birefringence axis.

**Figure 4:**
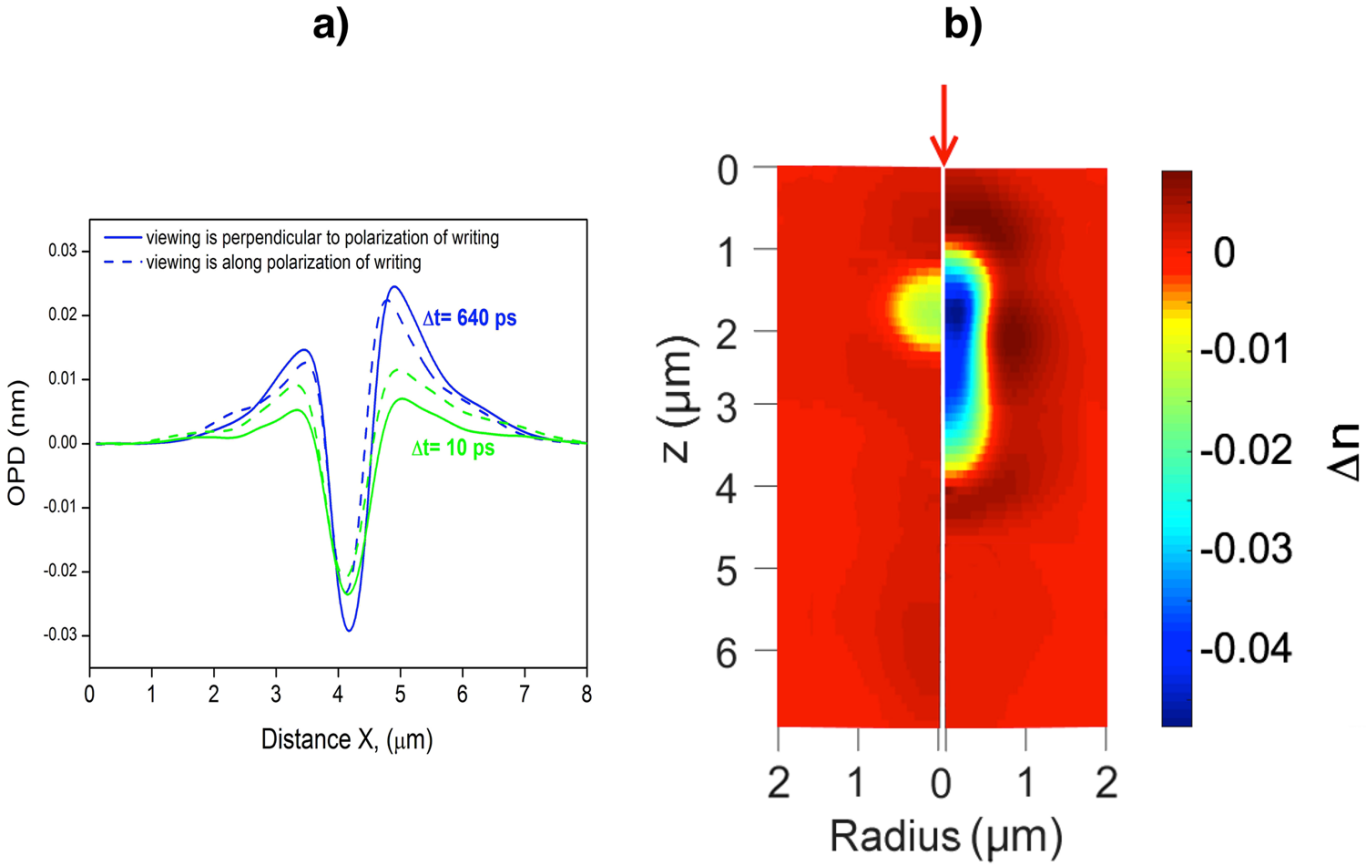
(**a**) Profiles of OPD for two orthogonal orientations of the dumbbell-like structure for the side view in the centre of a modification, produced by the burst with inter-pulse intervals Δ*t* = 10 ps and 640 ps, and b) 3D distributions of refractive index change Δ*n(r,z)* in the cylindrical coordinates produced by the original pulse (on the left, *Δt* = 0 ps) and a burst with pulse separation interval *Δt* = 640 ps (on the right). Single pulse/bust energy is 100 nJ. The arrow shows the direction of the laser beam.

All modifications in silica glass produced by bursts with pulse separation interval in the range 10–3000 ps have pronounced negative optical phase difference (OPD) *φ(x, z) − φ*_0_ for the side view in the centre of a modification. Typical profiles of OPD for two orthogonal orientations of the dumbbell-like structure relative viewing directions and two different inter-pulse intervals are shown in Fig. 4(a). The profiles for two viewing directions have very similar forms, and this indicates that the dumbbell-like structure and “ears” seen in Fig. 3 do not impact on axial symmetry of OPD. This allows applying the inverse Abel transform to reconstruct 3D distribution of refractive index change according to eqs (3) and (4). Typical 3D mapping of refractive index change *Δn(r,z)* in silica glass is shown in Fig. 4(b).

A single pulse produces modification of the refractive index with a recognisable profile elongated in the direction of the laser beam, which is strongly varying along this direction with change of sign located almost in the centre of the modification^11^. A burst with the same energy inscribes modifications having pronounced negative refractive index change along major part of the modification length. Maxima of positive changes in the refractive index are shifted to the modification boundary.

**Figure 5:**
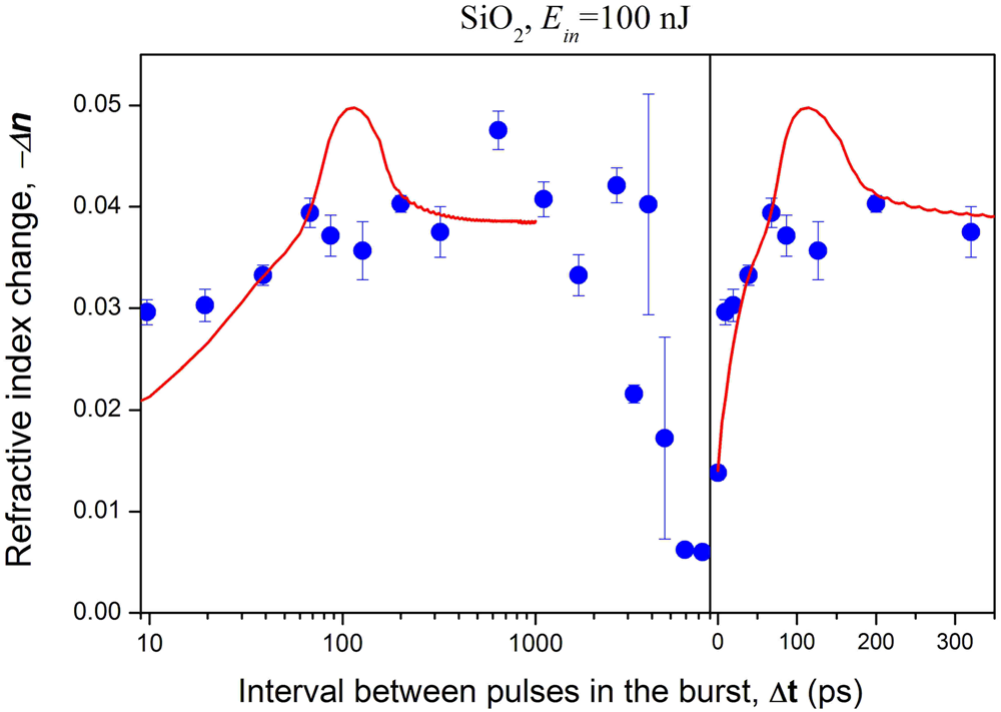
Experimental (blue points) and theoretical (red solid curve) plots for silica glass of peak refractive index change against burst pulse separation interval. Right and left pictures differ only by the time scales. The burst energy is 100 nJ. The experimental point with a zero interval corresponds to a single pulse with the same energy.

3D distributions of refractive index change in silica glass were built for series of modifications produced by bursts with various pulse separation intervals. The peak negative refractive index change was defined for each distribution, and results are summarised in Fig. 5. Each experimental point was obtained by averaging results of the treatments of six modification points: three for each polarization of the recording beam, these being along and perpendicular to the viewing direction. It was found an increase of the refractive index change upon pulse separation interval in the range of 0–100 ps, and a decrease on the scale of 4–8 ns with characteristic time of nearly 2 ns. In the range 0.5–4 ns there is considerable scatter of experimental points, which possibly has a periodic nature. Maximum magnitude of the refractive index change reached 0.05, exceeding the change produced by a single pulse by a factor of 4. The deviations of the experimental points from a monotone decay curve on a 1–8 ns time scale could be a manifestation of scatter of the beam waist location arising from fluctuations of the laser beam direction discussed in the Methods section. In any case dispersion of experimental points on the scale 1–8 ns does not influence the main results and conclusions.

**Figure 6:**
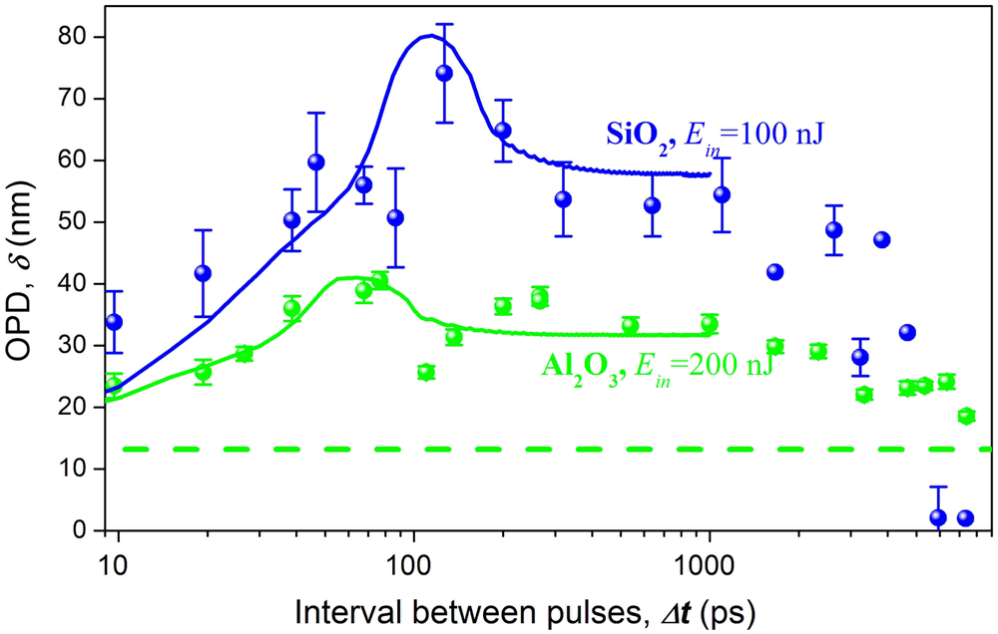
Experimental (points) and theoretical (solid curves) plots for silica glass modifications (blue points and curves) and sapphire (green points and curves) of peak OPD against burst pulse separation interval. Burst energy is 100 nJ for silica glass and 200 nJ for sapphire. The green dashed line shows the level of OPD in sapphire produced by a single pulse with the same energy.

For sapphire, the method of inverse Abel transform could not be applied for the available sample, because it had not a polished facet for the side view, and the only data collected was that of OPD. However, the response of sapphire can be analysed on the assumption that the correlation between OPD and refractive index change is the same for sapphire as for silica. Mapping OPD by viewing along the direction of the laser beam (top view) was obtained using QPM for the same modifications in silica glass, for which refractive index change were obtained using inverse Abel transform. As functions of the inter-pulse separation interval, the dependence of OPD for silica (Fig. 6) was found to be very similar to the dependence of the refractive index change (Fig. 5). Then mappings of OPD under the same conditions were carried out for modifications made in sapphire. A predominantly negative optical path difference in comparison with unmodified regions was found both for silica glass and sapphire, with a maximum magnitude at the centre of modification. The dependence of this magnitude on the pulse separation interval in the burst is shown in Fig. 6. Moreover, as functions of inter-pulse separation interval, the general character of the dependence of OPD for modifications in sapphire was found to be similar to that of fused silica, the only difference being that the magnitude of OPD reached its maximum at a pulse separation interval of 63 ps, which is less than for silica glass. The OPD produced by a burst is higher by a factor of 4 than the OPD produced by a single pulse with the same energy.

### Birefringence

**Figure 7:**
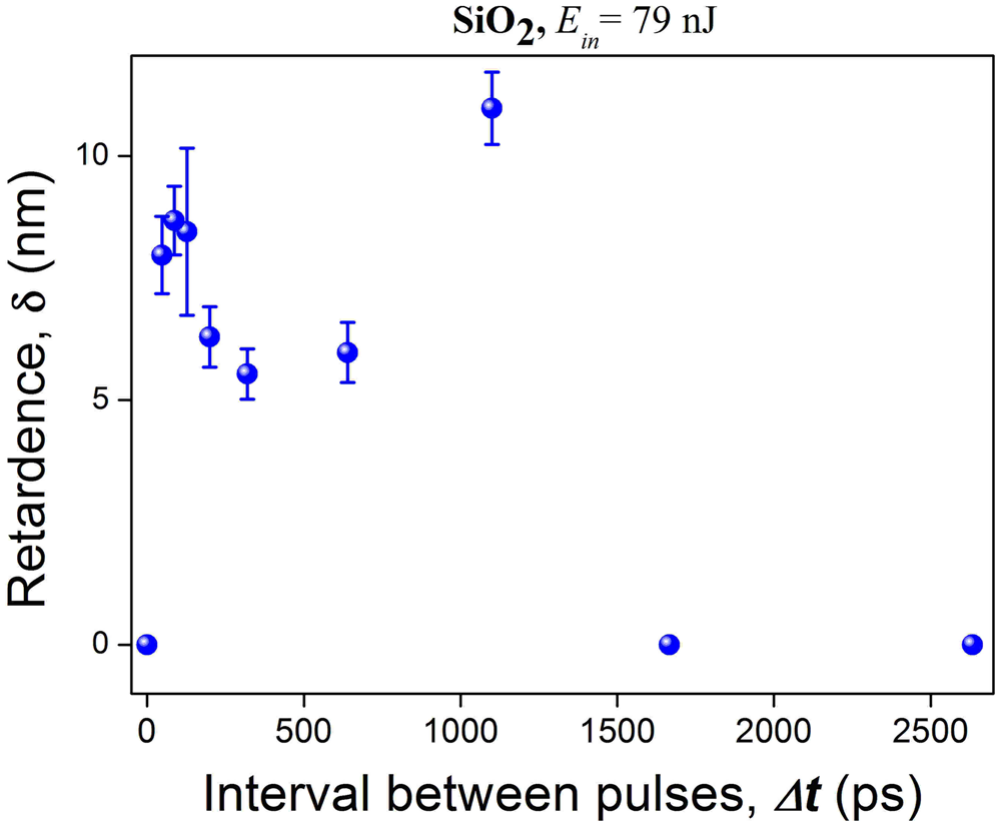
Plot of the retardance amplitude of the birefringent structure inscribed by a single burst in silica glass against pulse separation interval, with burst energy 79 nJ.

**Figure 8:**
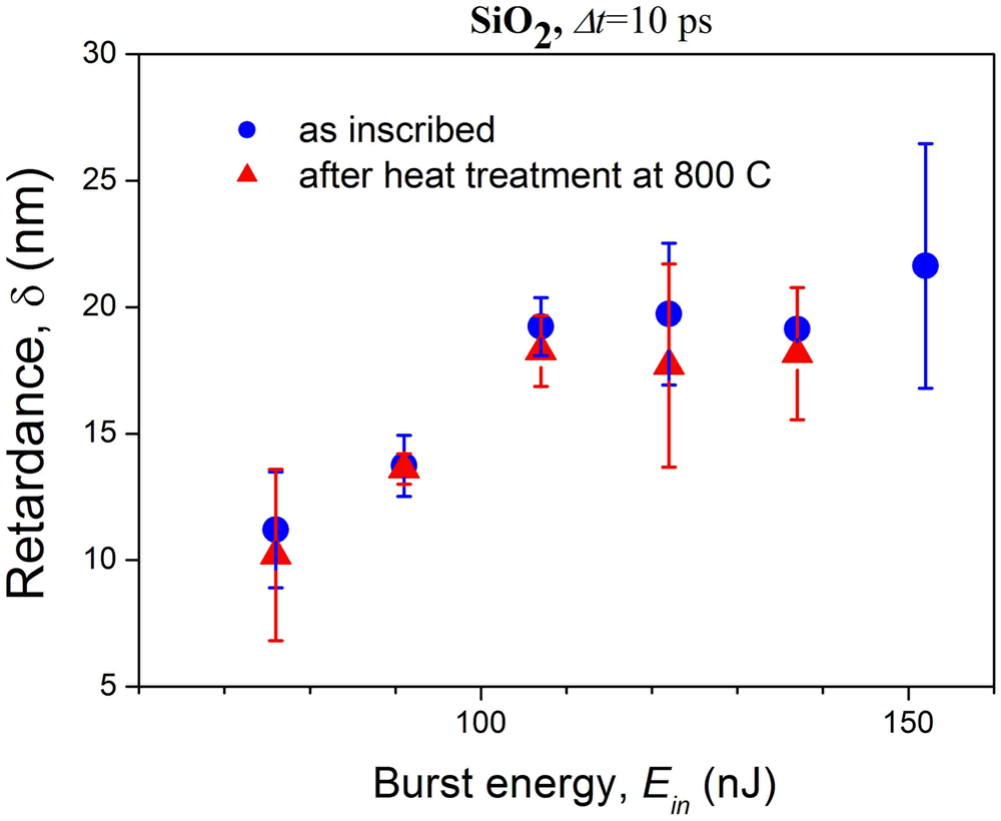
Plot for fused silica of retardance of the birefringent structure inscribed by single burst against burst energy for a pulse separation interval of 10 ps. Blue points are for measurements just after inscribing, red points are for measurements after a 10-hour heat treatment at 800 °C.

Dependence of the birefringence retardance upon the pulse separation interval in the burst resembles the dependence of refractive index change in silica glass. It has pronounced maxima at 95 ps and 1 ns for burst energy close to the inscription threshold energy, and becomes zero for higher pulse separation intervals, for which there is no modification (Fig. 7). The retardance slightly increases with the rise of burst energies and goes to zero at higher pulse separation interval retracing the dependence on the change in refractive index. There is no detectable birefringence for a single pulse (Fig. 3d). No degradation of inscribed birefringence was observed after a 10-hour heat treatment at 800 C (Fig. 8).

### Non-linear transmittance

**Figure 9:**
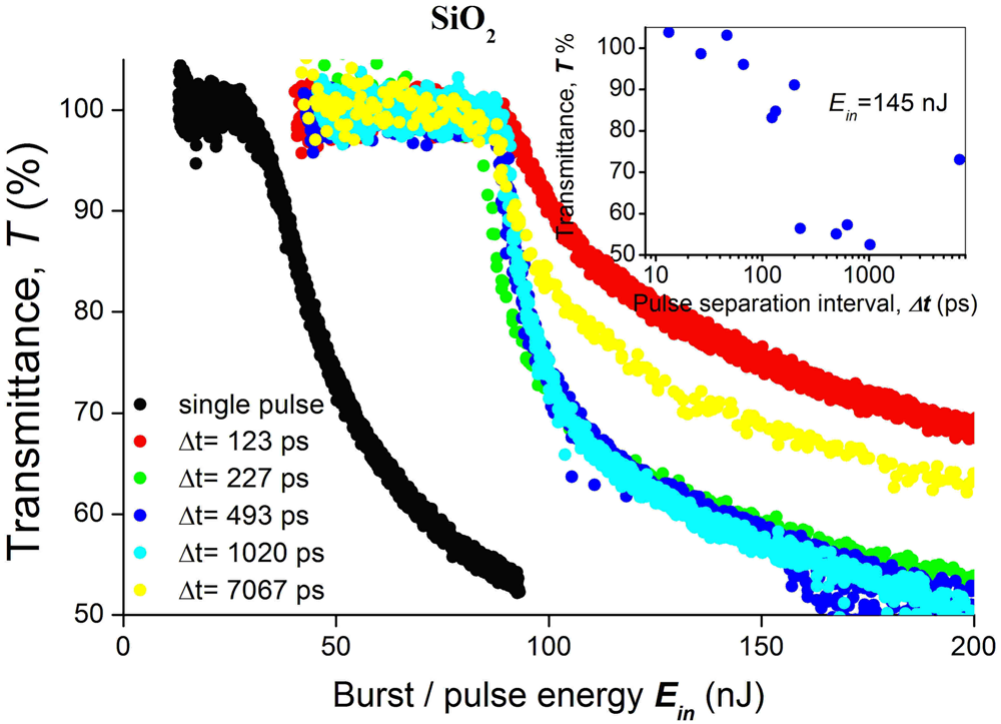
Plot for silica glass of pulse/burst transmittance against pulse/burst energy. Black points represent single pulses, coloured points represent bursts of varying inter-pulse intervals. The small inserted graph is a plot of the burst tail transmittance against inter-pulse interval with the burst energy of 145 nJ.

The dependence of single pulse transmittance on the pulse energy has a recognizable shape^12^, while the dependences of burst transmittance for the inter-pulse separation intervals 200–2000 ps are differed by steeper drops at energy close to the modification threshold (Fig. 9). It is important to note that the ratio of threshold energies for a burst and a single pulse is 2.4; that is, it coincides with the ratio of the burst energy to the energy of the first pulse in the burst. Dependence of transmittance on pulse separation interval *T(Δt)* could be expanded in two terms:

5 $$T({\rm{\Delta }}t)=\frac{{T}_{1}{E}_{1}+{T}_{bt}({\rm{\Delta }}t){E}_{bt}}{{E}_{in}},$$

where *T*_1_ is transmittance of the first pulse in the burst, *T*_*bt*_*(Δt)* is transmittance of the last part of the burst, that is, from the second pulse to infinity, *E*_*in*_ is energy of the burst, *E*_1_ is energy of the first pulse, *E*_*bt*_ is energy of the burst tail, that is, of the last part of the burst. The dependence of the burst tail transmittance *T*_*bt*_*(Δt)* on the inter-pulse interval was obtained from (5) taken into account that *E*_1_ = *E*_*in*_*/2.4* and *E*_*in*_ = *E*_1_ + *E*_*bt*_. The result for the fixed burst energy of 145 nJ is plotted in the insert of Fig. 9. This graph shows that the burst tail is not absorbed for the inter-pulse interval *Δt* not exceeding 40 ps. The transmittance of the burst tail decreases in the range 40–200 ps, reaches a flat region on the scale 200–1000 ps, and then rises for inter-pulse intervals of more than 1 ns.

## Discussions and Modelling

Femtosecond laser writing creates a permanent refractive index change in transparent dielectrics. This is initiated by multiphoton ionization, which is followed by linear absorption by the electron plasma and avalanche ionization^12,13^. In silica glass, over a time-scale of the order of 150 fs, free electrons and holes become locked to self-trapped excitons (STE)^12,14,15^, and so, for long pulses (>0.2 ps) STE efficiently absorbs laser pulses. In sapphire free electrons persist for nearly 100 ps, and it is expected that they provide strong linear absorption in this time interval^15^. Thus the observed modification increase on a time scale of 0–100 ps is inconsistent with known dynamics of electron excitations.

The role of the shock wave in the formation of voids was investigated in^5^, and it was shown that it produces rarefaction in the modified region which develops on a sub-nanosecond time scale. We interpret the dramatic increase in the change of refractive index, resulting from the controlled increase in the pulse separation interval from 0–100 ps, as an indication that in our experiments the role of rarefaction in the modification is significantly enhanced. We suppose that, if a laser pulse interacts with an already modified region at the same moment that rarefaction starts to develop due to the preceding pulse, it leads to the enhanced generation of stable defects, and thence to an increase in the change of the refractive index. We propose that in the first approximation this extra change can be described by the formula:

6 $${\rm{\Delta }}n({\rm{\Delta }}t)={\rm{\Delta }}{n}_{0}+\eta {\int }_{{\rm{m}}{\rm{o}}{\rm{d}}}\varepsilon ({\rm{\Delta }}t)dV,$$

where *ε(Δt)* is the first principal strain describing the largest deformation, *Δt* is the pulse separation interval in the burst, *Δn*_0_ is the change in refractive index produced by an ordinary single pulse with energy equal to the burst energy, *η* is the parameter of refractive index change enhancement due to rarefaction, and is material dependent. The integral is taken over the region of modification.

In order to verify the role of rarefaction we have built a model for dynamics of deformation in the modified region. The model is based on the motion equation for elastic media without energy dissipation describing propagation of elastic expansive waves^16^:

7 $$\frac{{\partial }^{2}\mathop{u}\limits^{\longrightarrow}}{\partial {t}^{2}}={c}^{2}{\nabla }^{2}\mathop{u}\limits^{\longrightarrow}-\frac{\nabla p(x,y,z)}{\rho },$$

where ***u*** is the deformation vector, *c* is the speed of expansive waves, defined by the formula:

8 $$c=\sqrt{\frac{E(1-\nu )}{(1+\nu )(1-2\nu )\rho }},$$

where *E* is Young’s modulus, *ν* is the Poisson ratio, and *ρ* is the media density. In formula (7) pressure *p* describes initial tensile stress, which instantaneously rises in the region of the electron–hole plasma produced by an infinitely short laser pulse. Alternatively the reader may find it helpful to think of the essence of the deformation dynamics if it is described in terms of strain. The instantaneous formation of electron-hole plasma and the consequent generation of electronic excitations change the interaction potential between ions of the media in such a way that just after laser excitation, the ion positions corresponding to zero strain are no longer equilibrium ones; that is, zero strain no longer corresponds to an equilibrium state. Then equation (7) describes relaxation of the initial zero strain field to the equilibrium strain field.

Eq. (7) was numerically solved by the finite element method by means of Comsol Multiphysics commercial code (version 3.5a), The Structural Mechanics Module. Our calculations covered a relatively short time interval of 1 ns. Thus we neglected heat transfer from the excited region, as it is a comparatively slow process. We simulated the geometry of the electronic excitation region by a prolate spheroid with symmetry axis coinciding with the direction of the laser beam. The diameter of the spheroid along the symmetry axis was taken to be equal to 4 μm, which is approximately the same as the longitudinal (i.e. along the laser beam) size of the modifications, and the transverse diameter was taken to be 1 μm. Distribution of the initial pressure ***p*** inside the spheroid was defined to be flat and equal to 0.1 GPa, although its magnitude does not qualitatively affect the result, because our model assumes only elastic deformations. The whole region of calculation was a sphere of radius 10 μm. Geometric and material parameters used in the calculation as well as the main results are collected in Table 1.

**Table 1:** Geometric and material parameters and results of numerical calculations of deformation dynamics.

	Semi-radiuses of the region of initial excessive pressure (μm)		Young’s modulus (GPa)	Poisson ratio	Density (g/cm^3^)	Delay for maximum tensile deformation (ps)
	***R***_***t***_	***R***_***l***_				
**SiO**_**2**_	0.5	2.0	73.1	0.17	2.203	**110**
**Al**_**2**_ **O**_**3**_			400	0.22	3.965	**63**

The calculated dynamics of the first principal strain together with formula (6) was used to approximate the dependence of the experimental refractive index change and of the OPD upon the pulse separation interval *Δ****t*** when only η was varied (Figs 5 and 6). The results of modelling show that the tension deformation increases during the first 63 ps and 110 ps for sapphire and silica glass respectively. This increase well describes the increase in refractive index change and OPD, and the model correctly represents the difference between glass and sapphire. The theoretical dependences have a specific upward excursion at the pulse separation intervals of 63 ps for sapphire and 110 ps for silica glass that are clearly observed in the experiment (Fig. 6). The transient process setting of the deformed state ends after around 300 ps.

Very similar results were obtained for density dynamics by solving the thermoelastic wave equation in spherical approximation for simulation of a transient lens induced by a femtosecond pulse in soda-lime glass^17^. In ref.^17^ substantially the same equation for transient elastic deformation was solved, although a different initial condition was used, namely temperature jump in a localized region, because temperature diffusion is rather slow for the time scale considered.

Thus, the calculated deformation dynamics agrees with the dependences of refractive index change and OPD for inter-pulse intervals in the range 0–300 ps. Evolution of the first principal strain in the cross section along symmetry axis in this time interval is demonstrated in Supplementary Video S1. Taking into account the close agreement between experimental and theoretical dependences, we assume that stretching of the crystal lattice or of the glass net promotes transformation of unstable electronic excitations into stable structural defects. Moreover, the tensile deformation accumulates and increases with each pulse of the burst. Efficiency of tension accumulation depends on the intervals between pulses. If a pulse comes at the moment of maximum tension, the resulting cumulative tension is maximized. Interestingly, just after this moment the elastic deformation wave becomes detached from the plasma zone, as shown by numerical calculations (Supplementary Video S1). A decrease in the refractive index change and OPD for a pulse separation interval larger than 3 ns is naturally explained by relaxation of electronic excitations and corresponding tensile stress, but this time domain is beyond the scope of our current model.

In addition to transient stretching of the modified medium there is another reason for the enhanced efficiency of a burst modification in comparison to that of a single pulse. Typical dependence of refractive index change upon pulse energy has a pronounced increase just above the modification threshold and then starts to saturate^18–20^. The saturation is connected with the intensity clamping effect caused by non-linear absorption in the electron-hole plasma^21^, restricting the density of the deposited energy. Therefore densities of structural defects, that cause refractive index change, do not increase with the increase of pulse energy. Instead, the volume of generating plasma is increased. The scenario is different for modification made by a burst. The results of non-linear transmittance measurements reveal that a necessary and sufficient condition for a burst modification is that the ionization threshold is attained with the first pulse, while energies of all subsequent pulses are insufficient for ionization. Indeed, the threshold energy for a burst is higher by a factor of 2.4 than for a single pulse; the same ratio as that between the burst energy and the energy of the first pulse in the burst (Fig. 9). This leads us to conclude that absorption of the burst tail is due to absorption by transient electronic excitation prepared by the first pulse, because pulse energy in the tail is lower than the modification threshold of unexcited media.

The temporal form of the burst helps to minimize the size of the electron-hole plasma region due to decrease of the first pulse intensity to the threshold intensity of multiphoton absorption. The subsequent pulses are absorbed by transient electronic excitation only in the localized region prepared by the first pulse. Thus the density of deposited energy is enhanced in comparison with single pulse writing, that is, the clamping effect is avoided. Moreover absorption of the subsequent pulses could be more efficient than of the first pulse, due to the lower number of photons required for generation of one free electron from the transient electronic excitations (colour centres). Thus a high density of stable defects and a large change in refractive index is achieved.

The assumption that the transient electronic excitations is responsible for absorption the burst tail and variation of glass density on nano-meter scale^22^ can explain poor repeatability of OPD in silica glass in comparison with sapphire that is reflected in large difference of error bars in Fig. 6. We think that the variation of glass density serves as seeding points for development of instabilities in the rarefaction dynamics that is attended with development of “hot spots” of electronic excitations. Namely, the first pulse creates non-homogeneous distribution of electron plasma and electronic transient centres. Spots of higher electronic excitation experience higher expansion, and then the second and following pulses enforce this inhomogeneity, as their absorption is higher are in the regions of higher concentration of the transient centres, which are preferably created in the expanding spots. Thus the positive feedback between absorption and expansion leads to instability as a general property of systems with a positive feedback.

Absorption efficiency in silica glass increases as the interval between pulses increases, as shown in Fig. 9. This increase was measured in the range of 50–1000 ps, which is not a match with the dependence of tensile deformation, however it follows quite well small refractive index increase in the range of 0.1–1 ns (Fig. 5). Moreover the absorption efficiency dependence on the inter-pulse interval roughly corresponds to observations made in double pulse experiments^7^, where this absorption was tentatively ascribed to unstable colour centres. Summarizing, we ascribe increased refractive index change due to burst writing by the combined effects of tensile stress and transient colour centres absorption.

The anisotropic nature of the tensile stress arising in the plasma region can explain the birefringence and enlargement of the region of modification along the direction of polarization of the femtosecond beam (Fig. 3). The anisotropy could be due to electrostriction, driven by a quasi-permanent field arising from non-linear frequency conversion of a femtosecond pulse light wave on the periphery of the plasma region.

A new method for obtaining birefringent structure in silica glass due to high thermo stability opens perspectives for the development of long-term optical memory. Compared with optical memory based on volume nanogratings requiring more than 10 pulses separated by intervals longer than 1 μs^23^, the new method has an almost 10 times faster writing speed, because a single laser shot produces a thermally stable birefringence structure. Finally, the sub-nanosecond burst offers an increase of throughput in ultrafast laser micromachining performance.

## Conclusion

We have proposed and demonstrated a method for efficient material modification with sub-nanosecond and nanosecond bursts of femtosecond pulses with decaying amplitudes in the burst. The burst activates an additional mechanism of femtosecond modification based on the simultaneous actions of rarefaction and electronic excitation. The innovative burst produces an enhanced refractive index change and a thermally stable birefringent structure even with a single laser shot, due to localization of energy deposition through a reduction in peak pulse intensity. Transient colour centres mainly control absorption of the second and all subsequent pulses in the burst, but the crucial role in modification enhancement belongs to the transient tensile strain accumulated by the repeated action of laser pulses. The largest enhancement of refractive index change in silica glass was obtained for pulse separation intervals in the range of 50 ps–3 ns.

## Electronic supplementary material

Video S1
