# Author Correction: Single shot laser writing with sub-nanosecond and nanosecond bursts of femtosecond pulses

**DOI:** 10.1038/s41598-018-22648-4

**Published:** 2018-03-12

**Authors:** Andrey Okhrimchuk, Sergey Fedotov, Ivan Glebov, Vladimir Sigaev, Peter Kazansky

**Affiliations:** 1 D. Mendeleyev University of Chemical Technology of Russia, Miusskaya square 9, Moscow, 125047 Russia; 2 Optoelectronics Research Centre, University of Southampton, Southampton, SO17 1BJ UK

Correction to: *Scientific Reports* 10.1038/s41598-017-16850-z, published online 29 November 2017

The Acknowledgements section in this Article is incomplete.

“Advanced Research Foundation, grant #7/019/2014-2017, supported the laser inscription experiments. Ministry of Education of Science of Russian Federation, grant #14.Z50.31.0009, have funded measurement, math treatment and analysis of the obtained data. The authors are grateful to Dr. R. Drevinskas for discussions of methods for measuring the optical phase.”

should read:

“Advanced Research Foundation, grant #7/019/2014-2017, supported the laser inscription experiments. Ministry of Education of Science of Russian Federation, grant #14.Z50.31.0009, have funded measurement, math treatment and analysis of the obtained data. A part of the study related to the measurements of refractive index change was also supported by the Royal Society (UK) International Exchanges Program (Grant IE150203). The authors are grateful to Dr. R. Drevinskas for helpful discussions.”

